# Aberrant Resting-State Brain Function in Adolescent Depression

**DOI:** 10.3389/fpsyg.2020.01784

**Published:** 2020-07-21

**Authors:** Ning Mao, Kaili Che, Tongpeng Chu, Yuna Li, Qinglin Wang, Meijie Liu, Heng Ma, Zhongyi Wang, Fan Lin, Bin Wang, Haixia Ji

**Affiliations:** ^1^Department of Radiology, Yantai Yuhuangding Hospital, Qingdao University, Yantai, China; ^2^Medical Imaging Research Institute, Binzhou Medical University, Yantai, China

**Keywords:** adolescent, depression, regional homogeneity, magnetic resonance imaging, resting state

## Abstract

To explore the changes of brain function and conduct clinical differential diagnosis based on support vector machine (SVM) in adolescent patients with depression. A total of 24 adolescent patients with depression according to CCMD-3 and DSM-5 and 23 gender, education level, body mass index, and age matched healthy controls were assessed with 17-item Hamilton Depression Rating Scale (HAMD). HAMD scores were requested from ≥17 of patients. Three−dimensional T1 and resting-state functional magnetic resonance imaging data were acquired from all participants. The data were analyzed using SPM 12 and REST1.8. Two-sample *t*-test was conducted to compare regional homogeneity (ReHo) values among the groups of participants. Finally, based on SVM classification, clinical differential diagnosis of the patients was carried out. The receiver operator characteristic (ROC) curve were used to confirm the performance of the SVM model. An increase ReHo values were observed in the lingual gyrus, middle occipital gyrus, postcentral gyrus, and precentral gyrus, whereas a decrease in ReHo was found in vermis compared with the control group. The SVM model showed good performance in classification prediction of adolescent depression, with an area under curve (AUC) of 0.778 [95% confidence interval (CI), 0.661–0.797]. The changes in the spontaneous neural activity of these regions may play an important role in the neuropathological mechanism of adolescent depression and may provide promising markers for clinical evaluation.

## Introduction

Depression during adolescence is a serious problem because of its high prevalence, the main manifestations of adolescent depression are sad mood, anxious, unpleasantness, lack of interest and motivation for entertainment and learning, a bad temper, easily irritable, or even suicidal (Cross-Disorder Group of the Psychiatric Genomics Consortium, 2013). Depression has a high suicide rate and the second highest disability rate in the world (Rosso et al., 2005; Jia et al., 2010; Ferrari et al., 2013). Adolescent depression affects the cognitive function of patients, and has a serious impact on the learning and life of adolescents (Den Hartog et al., 2003; Mao et al., 2016). Although knowledge about the etiology of depression has increased in the last decade, it is still difficult to explain and predict who becomes depressed, because of the many factors involved, and the diagnosis is mainly based on clinical symptoms. To reduce the huge burden on patients, their families and society, further research is needed.

Investigation of adolescent depression by functional MRI (fMRI) may reveal abnormal brain regions and help us to understand further the pathophysiology of adolescent depression. Regional homogeneity (ReHo) is a common calculation method used in functional magnetic resonance imaging and represents a certain similarity among different voxels in time series (Zang et al., 2004). The rest-fMRI is a powerful tool for assessing brain neural networks and activity (Mantini et al., 2007; Lee et al., 2013), which has been used for disruption of functional connectivity networks in depression. Previous studies suggested that brain dysfunction among depression patients is mainly observed in the limbic system (Fang et al., 2015). Furthermore, brain dysfunction is exhibited by reduced connectivity between the anterior cingulate cortex and left dorsolateral prefrontal cortex and bilateral amygdala (Deligiannidis et al., 2013; Cullen et al., 2014) and reduced posterior cingulate cortex-right amygdala connectivity (Chase et al., 2014).

However, few studies have conducted an examination of the functional alterations in adolescents suffering from depression. As a data-driven technique, machine learning plays an important role in analyzing neuroimaging data (Liu et al., 2015). Whether machine learning method can be used for classification prediction based on these changes is also unclear. Support vector machine (SVM) is a commonly used machine learning method for building a classifier. It aims to create a decision boundary between two classes that enables the prediction of labels from one or more feature (Hoekzema et al., 2017). In the present study, we used the ReHo methods to investigate the whole brain functional abnormalities and conduct clinical differential diagnosis based on SVM in adolescents diagnosed with depression.

## Materials and Methods

### Participants

We recruited 24 subjects (mean age: 17.31 ± 1.34 years; range 13–19 years) from the Department of Psychiatry in Yantai Yuhuangding Hospital of Qingdao University, Qingdao, China. All of the participants were diagnosed individually by two trained psychiatrists using the Structured Clinical Interview for Diagnostic and Statistical Manual of Mental Disorders-5 and 17-item Hamilton Depression Rating Scale (HAMD-17) score greater than 17 (Francis et al., 2010). Exclusion criteria included the following: (1) had a past and present medical history of psychiatric or neurological disorders and first-degree relatives; (2) had any severely unstable disease that requires medical treatment or hospitalization; (3) had a history of drug abuse or drug dependence within a year; (4) used hormone contraceptives; (5) was left-handed; and (6) had a history of craniocerebral trauma. Twenty-three healthy controls group who matched with the age, sex, body mass index (BMI) and education level were selected in the local community. The healthy controls group met all of the following inclusion criteria: (1) no history of depressive episodes; (2) no antidepressants and other antipsychotic medications. The healthy controls group did not meet any of the exclusion criteria, which are the same as the exclusion criteria of the depression group. This study was approved by the Ethics Committee of the Yantai Yuhuangding Hospital of Shandong Province, China. All subjects signed informed consent.

### Imaging Acquisition

Scanning was performed with a 3.0T MR (Skyra, Siemens, Erlangen, Germany) with a standard eight-channel head coil. The three−dimensional T1-weighted gradient echo pulse sequence parameters were as follows: TR/TE = 1940 ms/3.08 ms, flip angle = 15, FOV = 250 mm, and voxel dimensions = 1.0 mm × 1.0 mm × 1.0 mm. Resting state functional scanning was acquired using a gradient echo–echo planar imaging sequence. The scanning parameters were as follows: TR/TE = 2000 ms/29 ms, field of view = 192 mm, and voxel dimensions = 3.0 mm × 3.0 mm × 3.0 mm.

Resting-state fMRI data analysis was performed on SPM 12^1^ and the Resting State fMRI Data Analysis Toolkit (REST, V1.5_101101^2^). The basic steps of data processing were as follows. After the first 10 EPI data were removed, the time and head motion were corrected. Head-motion correction was carried out to artificially remove data whose head-motion was >2.0 mm and rotation was >2.0°. The remaining dataset was spatially normalized to the Montreal Neurological Institute template. Each voxel was resampled to 3 mm × 3 mm × 3 mm. Then, spatial normalization, De linear drift, extraction of low-frequency oscillating signal by 0.01–0.08 Hz and elimination of high-frequency noise. Using linear regression, several sources of spurious covariates along with their temporal derivatives, including the six head motion parameters, global mean, white matter, and cerebrospinal fluid, were removed. And consistency of time sequence of the brain voxel and peripheral 26 voxels were calculated to obtain Kendall’s coefficient of concordance (KCC), which is the ReHo value of the voxel. The spatial smoothness of the image is processed to reduce the difference brought by the individual with a 6 mm FWHM of smoothing kernel.

### Statistical Analysis

The statistical analyses were performed with SPSS 22.0. We conducted a two-sample *t*-test and χ^2^ test to examine demographic data (age, sex, BMI, education level, and length of illness) and HAMD scores among the patient and control group. Two-sample *t*-test was used to identify differences in brain ReHo across the entire brain of the participants (*p* < 0.05 had statistical significance). The volume of the region ≥10 (ReHo) voxels had statistical significance. SVM based on the functional abnormalities was used in classification prediction. The SVM model was applied by using the left-one cross-validation method. The receiver operator characteristic (ROC) curve were used to confirm the performance of the SVM model.

## Results

### Demographic Characteristics

Table 1 shows the demographic data of the subjects. No significant differences in age (*t* = −0.223, *p* = 0.996), gender (*t* = −0.523, *p* = 0.836), education level (*t* = 0.503, *p* = 0.657), and BMI (*t* = −0.056, *p* = 0.932) were found between the two groups (*p* > 0.05).

**TABLE 1:** Comparison of the general information between the patient and control groups.

	Control group (*n* = 23)	Patient group (*n* = 24)	*p*-Value
Gender (W/M)	12/11	14/10	0.836
Age (year)	18.21 ± 1.29	17.31 ± 1.34	0.996
BMI	23.3 ± 3.2	24.4 ± 2.5	0.932
Education level (year)	9.8 ± 3.2	10.1 ± 2.2	0.657
Mean duration of the illness (d)		4.20 ± 6.56	
HAMD		21.4 ± 3.09	
			

### Group Differences of Brain ReHo

**FIGURE 1:**
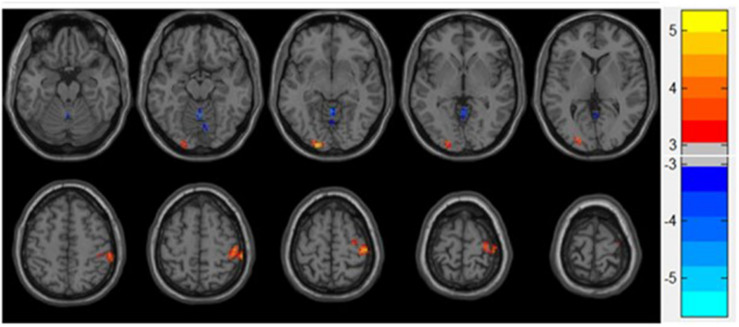
Statistical maps showing ReHo differences between patients and control group. Blue denotes decreased ReHo, and the color bar indicates the T-values from two-sample *t*-tests.

Increased ReHo was observed in the lingual gyrus, middle occipital gyrus, postcentral gyrus, and precentral gyrus, whereas reduced ReHo was found in the vermis of the patients diagnosed with depression compared with the control group. The results are shown in Table 2 and Figure 1.

**TABLE 2:** The difference of ReHo between the depression group and the control group.

	Cluster size	Coordinates (MNI)			AAL	T-Value	R/L
		*X*	*Y*	*Z*			
Lingual gyrus	22	−26	−96	−9	47	4.46	L
Middle occipital gyrus	15	−26	−96	−9	51	4.46	L
Postcentral gyrus	65	48	−24	63	58	4.35	R
Precentral gyrus	32	48	−24	63	2	4.35	R
Vermis_4_5	46	0	−51	−9	111	−3.55	

**p* < 0.05 (corrected), cluster size (voxel) ≥ 10, right/left (R/L).*

### SVM Prediction Results

**FIGURE 2:**
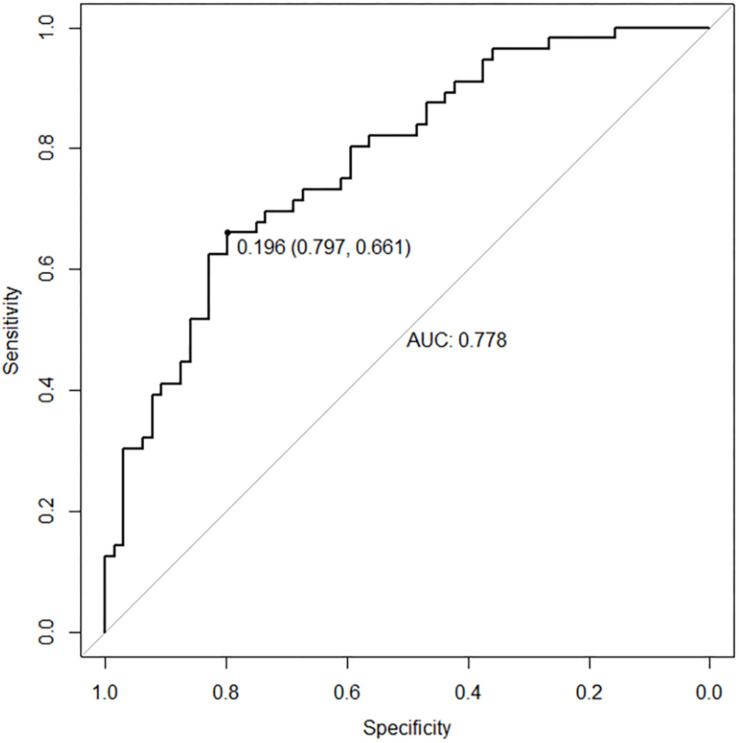
The ROC curve of SVM model. The SVM model showed good performance for the classification prediction of adolescent depression, with an AUC of 0.778.

As shown in Figure 2, the classification prediction of SVM is conducted based on the whole brain functional abnormalities. The SVM model showed good performance for the classification prediction of adolescent depression, with an area under curve (AUC) of 0.778 [95% confidence interval (CI), 0.661–0.797] (Figure 2).

## Discussion

This study was undertaken to elucidate the resting-state functional characteristic in the brains of adolescents diagnosed with depression before treatment. We found that the cerebral gray matter of the lingual gyrus, middle occipital gyrus, postcentral gyrus, precentral gyrus, and vermis of these patients was abnormal. This result was consistent with those of previous studies. We found three significant points in studying depression. First, depression patients had different ReHo values in some regions of the brain compared with the control group, suggesting that adolescent depression patients had functional abnormalities. Second, the SVM model showed good performance in the classification of adolescent depression by using different ReHo values as characters.

According to our study, the patients had increased ReHo in the left lingual gyrus compared with controlled participants. The lingual gyrus is part of the visual recognition network, which plays a role in the perception of facial emotion and is crucial for social functioning and individual emotion (Kop, 2012; Kong et al., 2015). A previous study found that the lingual gyrus is part of a “fear” network that regulates the processing of fear (Carlson et al., 2009). Therefore, any ReHo anomalies of major depression disorder in the visual cognitive circuit may be due to negative bias in emotion recognition and processing (Sun et al., 2018). In sum, this finding indicated that differences in ReHo in the lingual gyrus may represent abnormalities common to major depression disorder and adolescent depression.

Increased ReHo was observed in the left middle occipital gyrus, relative to healthy controls. The occipital lobe contains most of the anatomical area of the visual cortex, which helps to process visual information and communicate with the cerebral cortex and plays an important role in the perception of facial emotions (Teng et al., 2018). Consistent with previous studies (Guo et al., 2013), our findings showed that patients diagnosed with major depression disorder have increased activity in the left occipital gyrus compared with the control group. Depression patients with abnormal occipital brain activity show excessive attention preference for negative visual information (Guo et al., 2013). Depression-related occipital gyrus abnormalities are associated with abnormal neuropsychological activity, leading to motor block and decreased attention (Yu et al., 2017). In sum, these findings indicate that the middle occipital gyrus may play a key role in the pathophysiology of adolescent depression.

The finding of hyperactivity in the right pre- and postcentral gyrus in adolescent depression is convergent with the literature on the function of these brain regions. The pre- and postcentral gyrus show activation during emotion regulation (Domes et al., 2010). Previous structural evidence supports our findings of increased gray matter volume with regard to anxiety and depression in the postcentral gyrus of adolescents and adults (Wehry et al., 2015). Moreover, a positron emission tomography study further reported that anxious symptom improvement was related to reduced regional blood flow and serotonin synthesis rate in the postcentral gyrus in anxiety disorder (Frick et al., 2016). Major depression disorder causes lower brain surface area and gray matter volume in the precentral gyrus (Schmaal et al., 2017). In summary, the findings of this study may suggest an uncertain relationship between the central anterior and posterior central gyrus and the pathophysiology of adolescent depression. However, this study cannot explain that pre- and posterior gyrus hyperactivity is a feature or state imaging feature of depression. Thus, we need to examine and justify further this relationship.

An interesting finding of our study was the decreased activity in the cerebellum vermis in the patients group compared with the control group. The cerebellum, a structure that has an important role in the execution and planning of movement (Buckner, 2013) and is involved in emotional and cognitive processing, was reported in recent depression studies (Lin et al., 2012). The relation between cerebellum brain activity and depressive symptoms has been demonstrated by fMRI and sMRI findings (Xu et al., 2017). An increasing number of studies have focused on the role of the cerebellum in depression (Cheng et al., 2019), and our research suggests that the cerebellum vermis may be involved in the process of adolescent depression. However, this result needs further experimental verification.

The present study has several limitations. First, our sample size is relatively small and may thus limit statistical efficiency and the translational value of the results. Due to the small sample size, we did not verify the machine learning model with an independent dataset. Second, the cross-sectional design of this study prohibits causal interpretations of the results. Whether the abnormalities reported represent risk markers for depression or whether they emerge during the course of illness as a result of disease processes remains unclear. Large sample and longitudinal research are required to address these questions.

## Conclusion

The changes in the spontaneous neural activity of these regions may play an important role in the neuropathological mechanism of adolescent depression and may provide promising markers for clinical evaluation.

## Data Availability Statement

All datasets generated for this study are included in the article/supplementary material.

## Ethics Statement

The studies involving human participants were reviewed and approved by the Research Ethics Committee of the Yantai Yuhuangding Hospital of Shandong Province, China. Written informed consent to participate in this study was provided by the participants’ legal guardian/next of kin.

## Author Contributions

NM and KC designed the experiment, collected the data, performed the analyses, and wrote the manuscript. TC, YL, QW, ML, HM, ZW, and FL collected the data. BW and HJ contributed to the discussion and manuscript revision. All authors contributed to the article and approved the submitted version.

## Conflict of Interest

The authors declare that the research was conducted in the absence of any commercial or financial relationships that could be construed as a potential conflict of interest.

## Footnotes

1 http://www.fil.ion.ucl.ac.uk/spm/software/spm12

2 http://www.restfmri.net
